# Hyperuniform disordered terahertz quantum cascade laser

**DOI:** 10.1038/srep19325

**Published:** 2016-01-13

**Authors:** R. Degl’Innocenti, Y. D. Shah, L. Masini, A. Ronzani, A. Pitanti, Y. Ren, D. S. Jessop, A. Tredicucci, H. E. Beere, D. A. Ritchie

**Affiliations:** 1 Cavendish Laboratory, University of Cambridge, J. J. Thomson Avenue, Cambridge CB3 0HE, United Kingdom; 2 NEST, Istituto Nanoscienze – CNR and Scuola Normale Superiore, Piazza San Silvestro 12, Pisa, 56127, Italy; 3 Dipartimento di Fisica “E. Fermi” Universita’ di Pisa, Largo Pontecorvo 3, 56127 Pisa, Italy

## Abstract

Laser cavities have been realized in various different photonic systems. One of the forefront research fields regards the investigation of the physics of amplifying random optical media. The random laser is a fascinating concept because, further to the fundamental research investigating light transport into complex media, it allows us to obtain non-conventional spectral distribution and angular beam emission patterns not achievable with conventional approaches. Even more intriguing is the possibility to engineer *a priori* the optical properties of a disordered distribution in an amplifying medium. We demonstrate here the realization of a terahertz quantum cascade laser in an isotropic hyperuniform disordered distribution exhibiting unique features, such as the presence of a photonic band gap, low threshold current density, unconventional angular emission and optical bistability.

Periodic photonic systems, such as distributed feedback gratings (DFB)^1^, or, alternatively, two-dimensional photonic crystal (PC) designs have been thoroughly investigated in the recent years^2^,^3^,^4^,^5^,^6^,^7^. Aperiodic photonic systems, such as quasi-crystals^8^,^9^, offer a higher level of complexity, such as rotation symmetries prohibited by conventional periodic systems, and larger degrees of freedom originating from a richer Fourier spectrum. These features can be used for realizing aperiodic laser cavities, such as the Penrose lattice arrangement^10^,^11^. However, even though quasi-crystals lack translational symmetry, they still present long-range order and a quasi-periodicity. Random optical media^12^,^13^ have attracted an increased attention in both fundamental and applied research. Investigation of the complex scattering mechanisms which govern the light transport in such media is fundamental for the design of new synthetic optical materials. The importance of this research area stems from the investigation of fundamental topics such as light transport and localization in complex optical media and from the unique features offered by these distributions in different fields such as imaging through opaque media^14^, mainly for applications in biology, and harvesting of solar cell efficiency^15^. At the same time, disordered amplifying media offer unprecedented versatility since their optical properties can be tailored in order to achieve unconventional emission and unusual spectral characteristics. One of the most peculiar properties of these distributions consists in the possibility of engineering isotropic disordered media where the long-range order for long wavelengths is completely suppressed while the system still exhibits a photonic band gap (PBG)^16^,^17^,^18^. Such a counter-intuitive feature, the opening of a PBG without long-range order, is uniquely distinctive of this approach, in contrast to more conventional periodic and aperiodic systems. These PBGs can be tuned by acting on an order parameter which regulates the distribution properties and the balance between the local short-range order and the global disorder of the distribution. The disordered distribution presented in this work belongs to the so called stealth hyperuniform class^16^. The peculiar isotropic band gap offered by these distributions have been already object of investigation^16^,^17^,^18^,^19^,^20^,^21^, but it has never been implemented so far in a laser system. The importance of achieving lasing action in such disordered laser cavities resides in the possibility to overcome the limitations of angular and frequency emission offered by photonic crystal and quasi-crystal devices. By modifying the size and position of the scatters and by acting on the χ factor it is possible to have access to a virtual infinite number of combinations, covering in principle 4π stereo radiant angular emission and all the frequencies in the gain curve of the active material. THz quantum cascade lasers (QCLs)^22^ are compact, powerful sources employed in this spectral region for a huge variety of applications, ranging from metrology^23^ to sensing^24^ and spectroscopy^25^. The total modal confinement achieved in double metal QCLs renders these devices an ideal playground for the study of bidimensional disordered hyperuniform patterns. This work presents the investigation and realization of THz QC lasers based on a disordered, isotropic, hyperuniform structure. The QCL active region was processed into a disordered hyperuniform distribution of pillars, exhibiting a photonic band gap as large as 18% approaching the band gap of a conventional PC. The device lases on the localized modes at the lower edge of the band gap, which has been scaled in order to match the gain profile of the active region. The field emission presents unique features, such as optical angular emission bistability and low laser thresholds.

## Results

### Theoretical background and simulations

Our theoretical approach and procedure is based on the pioneering works of the Princeton group^16^,^17^,^18^ and is described in more detail in the Methods section. Disordered hyperuniform point patterns are characterized mathematically by reduced density fluctuations compared to a random system. This definition, which includes all the periodic, such as photonic crystals, and aperiodic, such as quasi-crystals, patterns also comprises a special subclass of random distributions. In the reciprocal space, this condition translates into the suppression of infinite wavelength fluctuations. Equivalently, the structure factor S(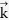), defined in Equation 1, tends to zero for wavenumber k tending to zero.


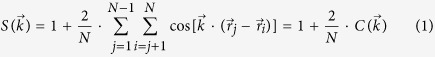


where N is the number of points and 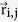 are the position vectors of the point distribution. In particular, we focused our attention to the stealth subclass of disordered distributions, which has a structure factor precisely equal to zero for a finite range of wavenumbers lower than a critical wavenumber k_c_. Among the many peculiarities of these distributions, the most fascinating and counterintuitive feature consists in the possibility to have a photonic bandgap even in the absence of long range order. Crystal and quasi-crystals which are deterministically determined by algorithms, exhibit sharp peaks in the diffraction patterns, which arise from long-range order and are ultimately connected to the structure factor S(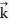) and to the Fourier transform of the geometrical structure in the real space. The opening of a band gap in disordered, even isotropic distributions characterized by the lack of long-range order results from the interaction between short-range geometrical order, hyperuniformity and Mie resonances of the scatters. Short-range order is related to the variance between the link lengths and interlink distances. The first one is a measure of the variance in the average distance between pillars. The interlink distance is defined as the variance in the distance between the midpoints of two neighbouring links. The imposition of the condition 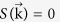 for 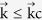 in the k-space corresponds to driving all the corresponding independent collective coordinates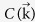, which represent a fraction of the total degrees of freedom of the system, to their minimum values. More details are reported in the Supplementary information. The distribution properties are better described and quantified by an order parameter χ which regulates the different optical regimes of the distributions and the degree of short-range geometrical order. The χ value, which is ≤1, is defined as the ratio between the constrained degrees of freedom to the total number of degrees of freedom of the distribution. Small values of χ correspond to more disordered distributions and do not yield sizeable PBGs, while for larger values of χ the optical modes tend to diffuse and the system starts developing long-range interactions, thus recovering the optical properties of periodic PCs. However, it is for intermediate χ values that these random distributions present unique features, such as sizable PBG in a disordered isotropic structures. The PBG angular isotropy in the reciprocal space is another important peculiarity achievable in these distributions, different from periodic and aperiodic structures.

**Figure 1:**
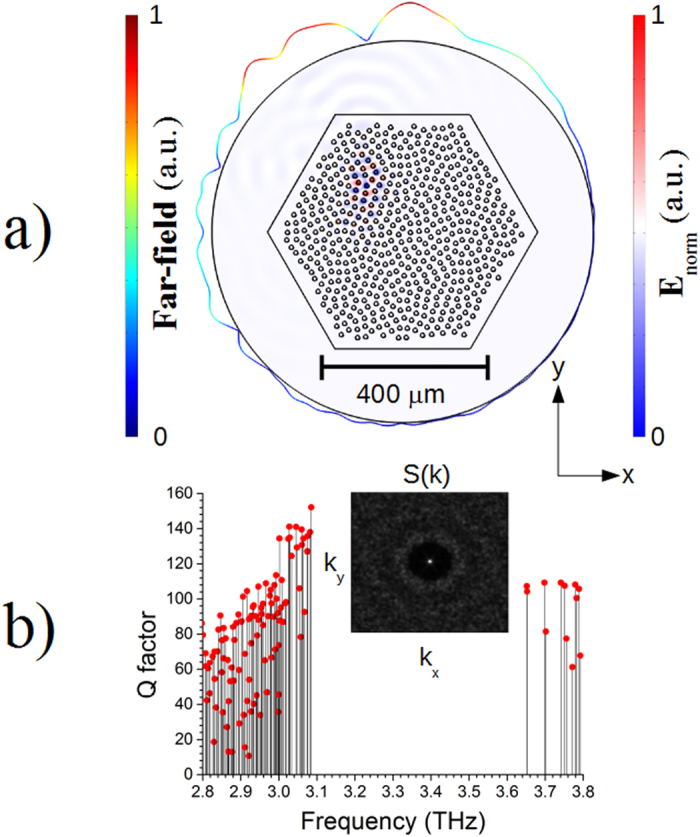
(**a**) Bidimensional finite element simulations performed with the commercial software Comsol Multiphysics of a hyperuniform disordered pattern with a χ factor = 0.5 arranged in a near hexagonal tile of high refractive index disks merged in a low refractive index matrix, which correspond to the active region and to the Cyclotene polymer substrate, respectively. This structure supports several localized modes. The highest frequency mode on the lower band edge on the PBG is reported together with the corresponding far-field. The mode is identified by the electric-field intensity in the AR, while the circumferential line’s radial deformation is proportional to the far-field intensity. (**b**) Q-factor for the different modes supported by this structure obtained from the eigenfrequency analysis. The inset reports the calculated structure factor S(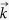) which is isotropic and presents an inner circle of S(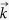) identical equal to zero for |k| ≤ k_c_ for some k_c_.

A bidimensional (2D) hyperuniform isotropic disordered disk pattern has been generated in a near hexagonal tile arrangement with a χ parameter equal to 0.5, which yields localized modes, with a sizable PBG. By varying the χ parameter it could be possible to modify the short-range order of the pillar distribution, e.g. the mode diffusion and observe the different optical regimes of these distributions^18^,^19^. This pattern was transferred in a commercial software based on the finite element method (Comsol Multiphysics v4.2), where the eigenmodes are simulated using the effective index approximation, in order to reproduce the optical modes and relative far-fields of the real device. Because of the almost total confinement of double metal THz QCL, a 2D approach based on the effective index yields a reliable approximation of the real device. The whole distribution has been scaled in order for the lower edge of the PBG to match the gain curve of the chosen laser active region (AR) emitting around 3.1 THz. The final 2D hexagonal distribution consists of a disk pattern each with a diameter of 11 μm and effective refractive index n_AR_ of 3.55 + i·0.01. These disks are merged in low dielectric constant material with a refractive index of n_BCB_ = 1.58 + i·0.0087, simulating the resin Cyclotene (BCB), which was used in the fabrication of the real devices in order to improve the mechanical stability. The eigenfrequency analysis reveals the emergence of a band-gap for TM polarized radiation as large as 18% related to the midgap frequency. The modes on the lower edge of the gap, centered around 3.1 THz, present in analogy with the modes of the standard photonic crystals, a maximum of the E-field in the high refractive index disks. Differently from those, though, they have a localized nature which is also a unique fingerprint of our approach. The modes lying on the upper band edge are dipole-like, e.g. they present a node in the E-field passing through the pillars, which, because of the reduced overlap between the electric field and the AR, makes them unsuitable for lasing. Examples of the modes lying on the upper band edge are reported in the Supplementary information. Noticeably, our approach allows us to obtain high optical mode Q factors, thus realizing non-conventional microcavities. These laser devices support a few distinct localized modes within the AR gain bandwidth, with peculiar frequency and angular emission characteristics. Among the several different optical modes supported by this structure, the localized ones lying at the lower border of the band edge emerge with a Q factor as high as ~150. According to the simulations performed with Comsol Multiphysics, the main limitation to the total Q factor is given by the radiative losses of the in plane component of the electric field. Reducing the radiative losses in this optical cavities cannot be achieved by increasing the periods of photonic crystals scatters around standard microcavities^26^. It could be possible to act of the scatters’ sizes and positions and on χ factor as well, in order to obtain more confined distributions, however this operation would require a re-design of the whole distribution. From an experimental point of view then, a further reduction in the radiation out-coupling would strongly affect the total emitted power and consequently the device performance’s characterization, e.g. the far field. The highest frequency mode on the lowest band-edge is shown in Fig. 1a together with the corresponding far-field. The PBG is also reported for completeness in Fig. 1b where the inset shows the corresponding isotropic structure factor S(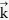). The mode presented in Fig. 1a has a Q-factor as high as 152 and the highest mode overlap with the active region, as better explained and quantified in the supplementary information. Based on the aforementioned characteristics and on the far-field analysis, it is assumed that lasing action will take place on the higher frequency modes lying on the lower band edge.

### Device fabrication

**Figure 2:**
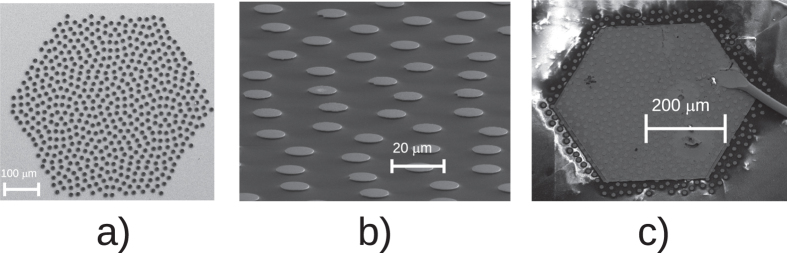
(**a**) SEM picture of the pillar disordered pattern after the ICP-RIE process. (**b**) SEM picture of the structure after having spun and thermally cured multiple BCB layers. The BCB was then etched in a second RIE process in order to expose the top of the pillars. (**c**) Final device mounted onto a copper block and wire bonded.

An 11.3 μm thick, high-doped (Si doping 6·10^16^ cm^−3^) version of the bound-to-continuum design reported in ref. 27 emitting around 3.2 THz has been used as active region for the device fabrication. The details of the fabrication process are reported in more details in the Methods section. A few typical scanning electron microscope (SEM) pictures reporting the most critical steps in the fabrication are presented in Fig. 2. The hyperuniform disordered disk pattern was transferred on the top surface of the active region through standard optical lithography. The photoresist mask was then etched through the active region by using an inductively coupled plasma reactive ion etching (ICP-RIE) process, which ensured vertical sidewalls, as shown in Fig. 2a. The following critical step, after having removed the photoresist left on top of the pillars, consisted in the spinning of multiple BCB layers, thermal curing and planarization. The polymer Cyclotene (3022–46 from Dow Company), which was already successfully employed in the fabrication of standard photonic crystal QCLs^5^, was chosen as the low refractive index material because of its low losses in the terahertz range and its excellent thermal and mechanical properties. A second RIE process was required in order to expose the pillars heads, as showed in Fig. 2b, to allow, by using photolitographic masking, metallic thermal evaporation and lift off, the realization of a top contact. Finally, the device after a final step of substrate thinning to ~200 μm, was mounted onto a copper block and wire bonded, Fig. 2c.

### Measurements

**Figure 3:**
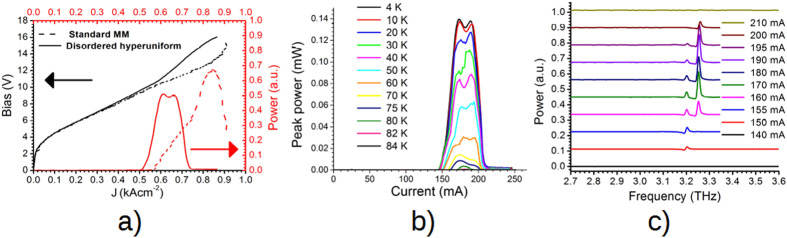
(a) Voltage-light-current characteristics of the disordered hyperuniform laser compared to a standard MM laser operating in the same condition of 5% duty cycle, 100 kHz repetition rate at 5 K. The current density of the disordered hyperuniform device has been manually scaled in order to reproduce as close as possible the curve trend of the MM laser below threshold. The threshold current density J_th_ is reduced from 575 Acm^−2^ for the MM laser to 504 Acm^−2^. (**b**) Temperature performance of the disordered hyperuniform laser. The maximum emitted peak power is ~ 0.14 mW c) Spectral analysis which reveals emission on two lasing modes. The two circles highlight the single mode biasing condition used in the far-field analysis.

A few hyperuniform disordered lasers were tested on the cold-finger of a continuous flow He cryostat and driven in pulsed operation mode with a repetition rate of 100 kHz and a duty cycle of 5% with the power measurements taken by a Golay cell having a Winston cone in front of the aperture, and a lock-in amplifier. They all showed consistent power, far field emission and current density characteristics. Moreover, in order to perform a direct comparison, a final 0.55 mm long, 140 μm wide QCL laser was fabricated from the same active region with a standard metal-metal (MM) wet-etching process. The results and the comparison are presented in Fig. 3. The MM emitting area is calculated from the measured device length of 0.55 mm and the average between the top and bottom ridge width. The exact calculation of the threshold in these devices is a non-trivial task due to the current spreading and uniformities, as already reported in refs 5 and 28. A determination of the current threshold assuming the nominal effective area of the active region, similar to what is reported in ref. 5, is shown in the supplementary information. This procedure yielded an upper limit in the reduction of the threshold current, around 56%, but does not match exactly the transport curves of the standard MM and hyperuniform laser. Consequently, Fig. 3a reports a comparison between the current densities of the two devices obtained by attributing an effective area to the hyperuniform laser given by the area that best reproduced the VI curves below threshold. This operation yielded a more conservative reduction in threshold, around 85%, from 575 Acm^−2^ for the MM laser to 504 Acm^−2^ for the hyperuniform one. This result is in good agreement with the 17% reduction in threshold presented in ref. 5. Because of the localized nature of our optical modes it would be expected an improvement with respect to the delocalized optical mode of conventional PC lasers^5^. Furthermore, such reduction corresponds to have contacted 65% of the nominal area, which is in clear contradiction with the evidence of Fig. 2c. Therefore, this value is identified as an upper limit to the density threshold. It is worth also mentioning that three different working devices exhibit similar thresholds. The threshold measurements of a standard MM QCL fabricated with the same active region is the lowest among three different devices tested. Since the diameter of the pillars is a few microns, the electronic transport should in principle be the same as in the bulk material. However, the presence of an external dead layer in the pillars because of the etching process, a non-perfect lithographic process and an imperfect biasing of all the pillars in the structure can affect the measured transport curve. Finally, since electronic transport takes place in all the pillars but only some of them are emitting, it is non uniform and a direct comparison with standard MM cannot be performed exactly. The reduction in the threshold current density, as shown in Fig. 3a, is due to multiple factors. It can partly be attributed to the more favourable mode distribution in the AR. In fact, the maximum of the E-field is concentrated in the AR patterned pillars, while the antinode of the in-plane E-field, which does not yield any contribution to the overall gain, is mostly located in the BCB region, which presents lower losses. Uniquely, the modes in this disordered structures present high Q factors, which significantly contribute to the threshold reduction, compared to standard Fabry-Perot or PC structures due to their localized nature and therefore to a better confinement of the optical modes. The nature itself of the mode localization differs from standard microcavities, e.g. defects in PBG arrangement. The observed slight reduction in the dynamic range of this laser compared to the standard MM one is due to the non-optimal overlap in frequency between the lasing modes and the peak of the AR gain curve. The temperature characterization of the hyperuniform disordered laser is reported in Fig. 3b. These lasers work up to 82 K, in good agreement with the 85K measured for a standard MM QCL. The wall-plug efficiency of the hyperuniform disordered laser is calculated to be 0.07% which is about 3 times lower than in a standard MM laser. This reduction is attributed to the fact that while the current is flowing through all the pillars contacted in the structure, the optical lasing modes overlap only with a part of them.

**Figure 4:**
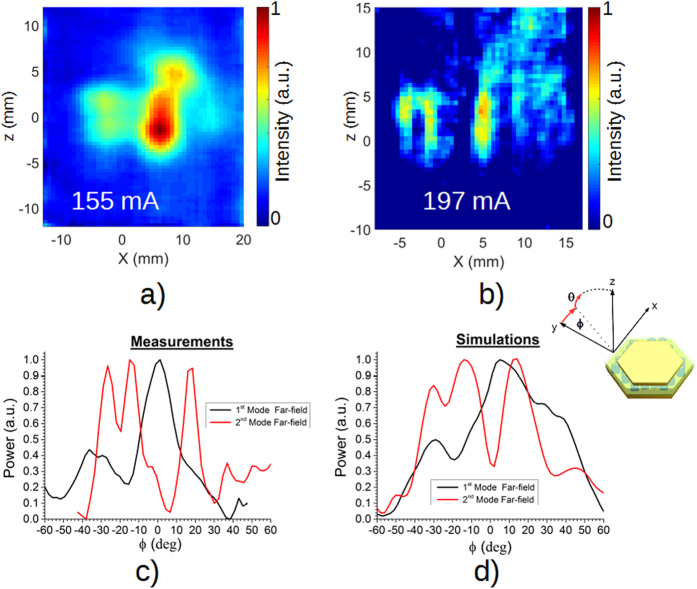
Far-field analysis of the device driven in the same operating conditions of Fig. 3(a). The far-fields (**a**) and (**b**) corresponds to a current of 155 mA and 197 mA flowing through the device, respectively. The intensity in the far-fields (**a**) and (**c**) have been averaged along the z-direction, in order to compare the measured far-field profiles (**c**) with the simulated ones (**d**).

These structures support several localized modes, all having peculiar angular emission and frequency fingerprints. A few optical modes overlap with the same active region, which prevents them from lasing simultaneously. The positioning of the bonding wire also affects the optical modes, by locally increasing the losses. Finally, the positioning of bonding pads makes a complete mapping of the mode emission difficult for these devices. A spectral analysis was performed using a Fourier transform infrared spectrometer from Bruker (model IFS66VIS) and a He-cooled bolometer as detector. As it is shown in Fig. 3c, this device supports two lasing modes emitting at 3.2 and 3.25 THz, respectively. The discrepancy of about 100GHz between the simulated lasing modes and the measured ones is attributed to the non-ideal shape and size of the final pillars. It is worth mentioning that a second identical device also presented consistent spectra, thus confirming the reproducibility of these measurements. It should be possible in principle to observe the spectral chaotic behaviour and the dynamic fluctuations typical of random lasers in a range of biasing conditions^29^ in this device. However, such an intriguing possibility requires a fast acquisition of the spectral emission, which could be achieved by using superconducting mixers or Schottky diodes, but cannot be performed by using Fourier transform infrared spectrometer and bolometer detection, requiring a few averages and characterized by typical acquisition times of a few minutes. Furthermore, the spectra reported in Fig. 3c are mostly intended as a biasing map in order to identify the more stable and favourable single mode emission conditions. The identification of the laser modes requires a far-field analysis and comparison with the simulated structures. Consequently, several far-fields were acquired with the axis orientation reported for clarity in the inset of Fig. 4d in the same biasing conditions reported in Fig. 3a at different operating currents. The far-fields were acquired using a Golay cell placed at a distance between laser and detector of 32 mm. An aperture with a 1.5 mm diameter was placed in front of the Golay in order to increase the spatial resolution. Figure 4a,b show the far-fields acquired with a device driving current of 155 mA and 197 mA, respectively. Figure 4a,b correspond to the far-field of the device emitting in a single mode according to the spectra presented in Fig. 3c. In order to perform a direct comparison between simulations and measurements, the far-fields of Fig. 4a,b have been averaged along the z-direction and normalized. In fact, the distinct angular features peculiar for each mode, which together with the emission frequency allow mode identification, are clearly distinguishable along the x-direction in Fig. 4a,b. Conversely, the intensity profile along the z-direction is mainly due to the highly diffracted non Gaussian-like divergence from subwavelength MM plasmonic features. Therefore, the mode profile along the z-direction yields poor information about the angular dispersion of the modes. The averaging over the z-direction, permits also a direct comparison between the 2D simulated far-field profiles and the 3D acquired ones. The far-fields have been recorded in Cartesian coordinates and translated into spherical coordinates considering that for relative small angles the approximation is within the experimental errors of these measurements, and because the far-field set-up available in the laboratory is designed to measure vertically emitting devices. The measured profiles are reported in Fig. 4c and show a very good agreement with the simulated ones, reported in Fig. 4d. The far-field of the second, higher frequency mode is the one already shown in Fig. 1a, while the second one is reported in the Supplementary information.

## Discussion

The optical bistability, the possibility to have completely different angular emission depending on the device biasing condition, is one important distinct feature of our approach which could be exploited further in other device architectures. The realization of different QCLs with different χ factors would allow the investigation of different optical regimes in random media^18^,^19^. Furthermore, by opportunely engineering the degree of disorder in the system, it could be possible to have in the same device modes and distributions with different degree of localization. This opens the way to the realization of an innovative type of waveguides and of optical light diffusion, where drastic changes of the emission properties in the material can be induced by acting on the device biasing, modifying the optical regime of the system.

In conclusion, we have demonstrated the first realization of lasing action in an isotropic, disordered hyperuniform pattern exhibiting a PBG as large as 18%. This result has been accomplished by realizing a THz QCL based on a pillar distribution etched in a 3.2 THz emitting active region. A protocol for the design of hyperuniform distributions was successfully developed and the final pattern was investigated and simulated with finite element method analysis. Simulations and measurements confirm lasing action in localized optical modes located at the lower edge of the photonic band gap. This result paves the way to the realization of non-conventional mode engineering and laser devices with respect to conventional PC and quasi-crystal lasers.

## Methods

### Fabrication

The hyperuniform disordered pattern was transferred on top of the AR by optical lithography using the positive photoresist S1828 from Shipley. This produced an optical mask of photoresist pillars with a thickness of approximately 2.6 μm. The ICP-RIE process was performed by using an Oxford Plasmalab-80 system and etched the 11.3 μm in 192 sec using an 8/10/6 BCl_3_/Cl_2_/Ar at 15 °C temperature with 0.2 Pa pressure and 25W/100W power. The BCB planarization process was based on Cyclotene (3022–46 from Dow Company). The polymer was spun at 4000 rpm and then heated up at 150 °C for 3 hr. A second controlled step of thermal curing up to 210 °C for a 2 hr 30 m was performed in an annealer operating in a N_2_ purged environment in order to allow multiple layer spinning. A similar final step of BCB spinning and thermal curing up to 250 °C was required in order to completely cover the pillar distribution and to obtain a chemically and mechanically stable film. The BCB layer was then etched using a Plasma-Pod RIE machine from JLS Designs, with 75 W power, a mixture (60–40%) of CHF_3_/O_2_ and a pressure of 60 sccm for 330 sec until the pillars’ tops are exposed by ~200 nm.

### Design calculate

The design of the hyperuniform pattern follows closely the procedure reported in^17^,^18^ starting from an N point distribution in a supercell based on a randomly perturbed triangular lattice of non-overlapping circular disks. The total potential energy Φ(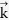) of the system is related to the C(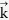) function previously defined in the main text, via an interacting pair potential with Fourier transform V(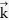) and is given by Equation 2:


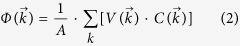


A is the area of the supercell in the direct space. Considering a cut-off wavenumber radius k_c_ in our system, the set of wave vectors 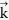 with |k| ≤ k_c_ is 2M(k_c_), where M(k_c_) is the total number of independent collective coordinates C(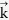) within the radius k_c_, which are constrained to their minimum. The χ parameter is defined as the ratio of the constrained degrees of freedom to the total number of degrees of freedom. In two dimension systems this corresponds to χ = M(k_c_)/2N ≤ 1. If the potential V(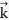) is assumed to be a positive constant value for |k| ≤ k_c_ and 0 otherwise, a minimization of the total potential energy Φ(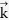) corresponds to a minimization of C(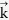) to a global minimum yielding stealth ground states within the radius k_c._

## Additional Information

**How to cite this article**: Degl’Innocenti, R. *et al*. Hyperuniform disordered terahertz quantum cascade laser. *Sci. Rep*. **6**, 19325; doi: 10.1038/srep19325 (2016).

## Supplementary Material

Supplementary Information
