# Supplementary material for: Hyperuniform disordered terahertz quantum cascade laser
Source: Sci Rep. 2016 Jan 13;6:19325. doi: 10.1038/srep19325 (PMC4725355; doi:10.1038/srep19325)
Supplement: Supplementary Information [file srep19325-s1.pdf]

Supplementary information for the manuscript

"Hyperuniform disordered terahertz quantum cascade laser"

R. Degl'Innocenti[a], Y. D. Shah[a], L. Masini[b], A. Ronzani[b], A. Pitanti[b], Y. Ren[a], D. S. Jessop[a], A. Tredicucci[c], H. E. Beere[a] and D. A. Ritchie[a]

[a]Cavendish Laboratory, University of Cambridge, J. J. Thomson Avenue, Cambridge CB3 0HE, United Kingdom; [b]NEST, Istituto Nanoscienze – CNR and Scuola Normale Superiore, Piazza San Silvestro 12, Pisa, I-56127, Italy; [c]Dipartimento di Fisica "E. Fermi" Universita' di Pisa, Largo Pontecorvo 3, 56127 Pisa, Italy

The highest frequency mode lying on the band gap lower edge is reported in Fig. 1 a). The second and the third highest

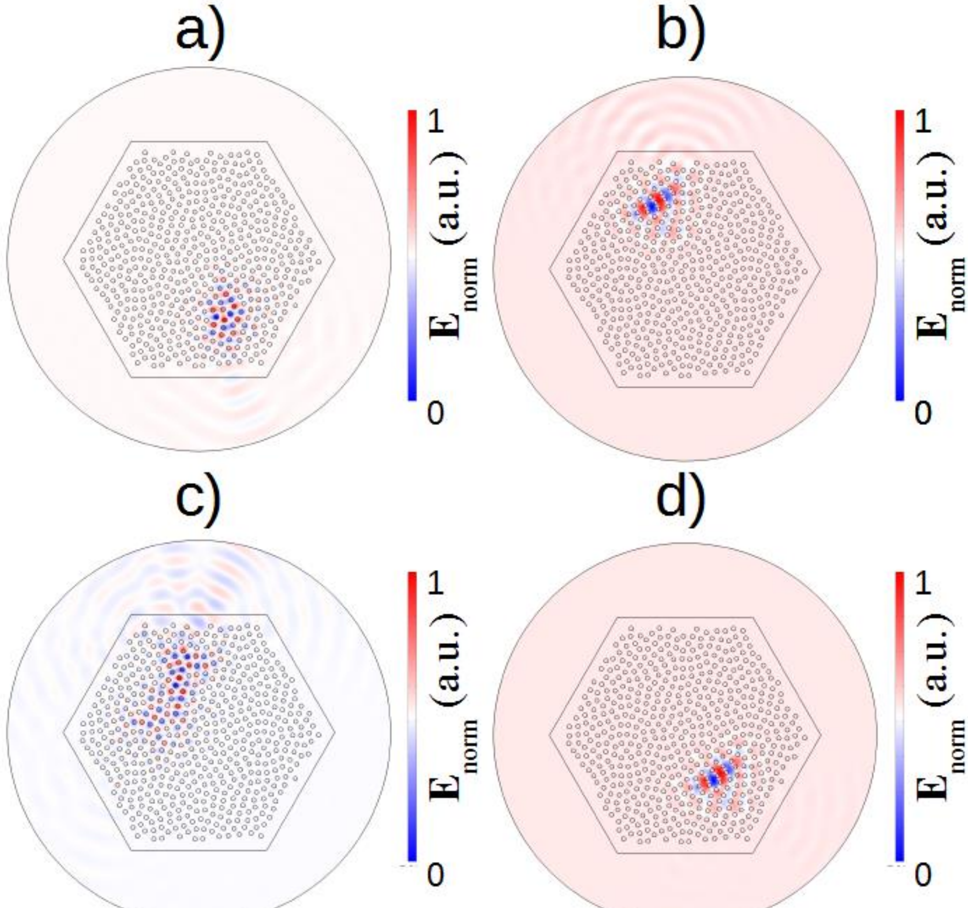

Fig. 1S. Optical modes supported by the hyperuniform disordered THz QCL calculated with the commercial software Comsol Multiphysics. All the E-field intensity have been normalized to unity. The optical modes a) and c) represent the second and third higher frequency modes lying on the lower edge of the PBG, respectively. The first two lower frequency modes lying on the upper band edge are shown in b) and d).

frequency modes are reported in Fig. 1S a) and c) respectively. In particular, Fig. 1S c) corresponds to the second lasing mode of Fig. 4 b). None of the different lasing devices presented emission on the mode of Fig. 1S a). This has been attributed to the positioning of the bonding wire which locally affects the device. The two lowest frequency modes

localized on the upper band edge are reported in Fig. 1S b) and c), respectively. These optical modes present a Q factor of ~ 100, significantly lower than the ones lying on the lower band-edge. The reduced overlap of these optical modes, mostly concentrated in the BCB region, with the active region make them unsuitable for lasing. In order to better quantify the overlap of the different modes supported by these device, we calculated the overlap integral Γ of the E-field with the active region, which quantifies the confinement factor of the E-field E(x,y) in the pillar area. The overlap integral is defined as the electrical energy density integrated over the active region normalized to the electrical energy density integrated on the whole space:

$$\Gamma = \frac{\left(\iint_{AR} \varepsilon(x,y)\cdot\left|E(x,y)^2\right|\right)}{\left(\iint_{\infty} \varepsilon(x,y)\cdot\left|E(x,y)^2\right|\right)}$$

where ε is the dielectric constant.

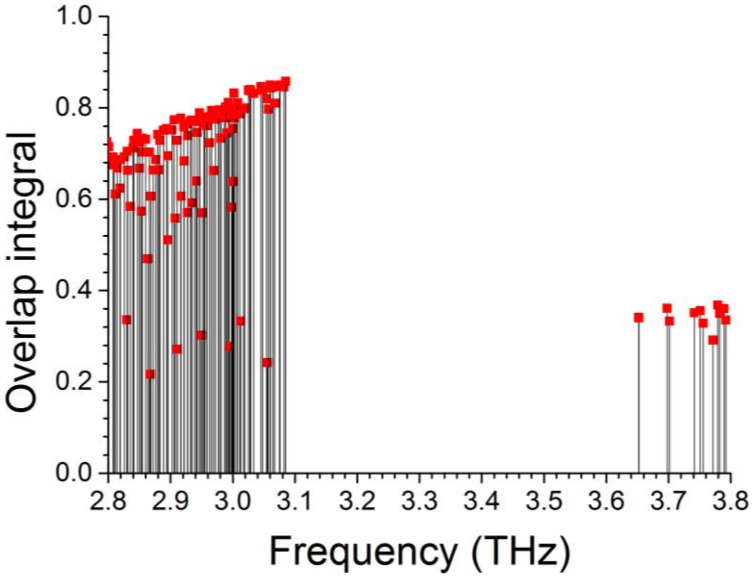

Fig. 2S. Overlap integral for the optical modes supported by the laser device.

As it is shown in Fig. 2S, the overlap integral is as high as ~ 85% for the modes lying on the lower edge of the PBG and ~ 35% for the ones lying on the upper band edge. Finally we report for completeness in Fig. 3S the spectrum corresponding

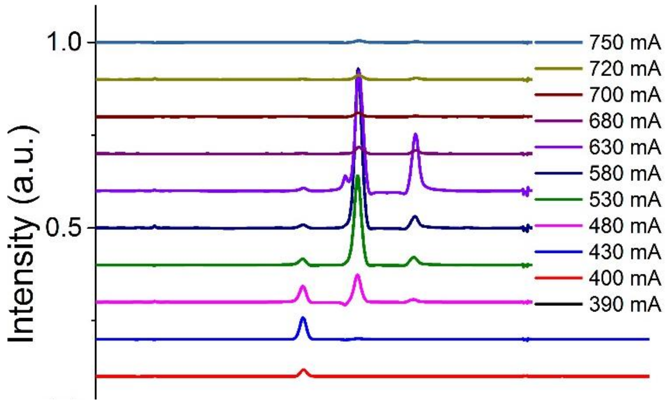

Fig. 3S. Spectral characterization of the MM QCL laser reported in Fig. 3 a). The spectra were acquired at different operating currents

to the MM THz QCL reported in Fig. 3 a). The device was operating in the same bias configuration and the spectra were acquired by using a Bruker spectrometer and bolometer detection, as for Fig. 3 c). The emission spans ~ 200 GHz, which can be attributed also to the limited length of the laser cavity. It is worth mentioning that two other lasers fabricated from the same active region, exhibited similar but slightly higher threshold currents, thus confirming the reproducibility of these measurements.

The threshold density reported in the main text in Fig. 3 a) represents a lower limit in the threshold reduction. An alternative procedure in order to obtain the current threshold considers the nominal active region area of the device [5] and is shown in Fig. 4S where the same curves of Fig. 3 a) are shown but scaled according to the recalculated area. This procedure yields

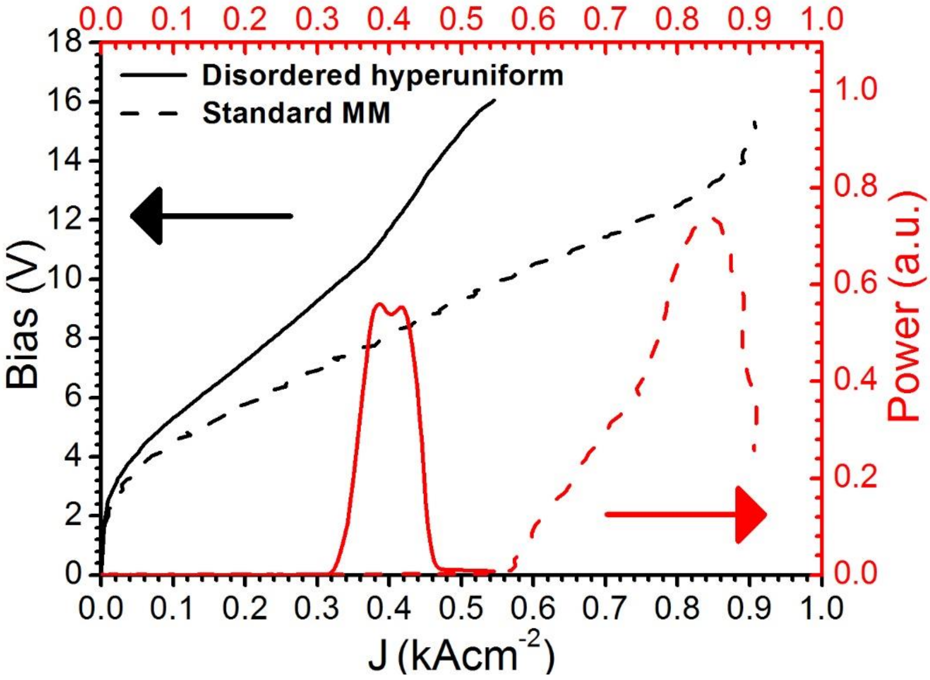

Fig. 4S Voltage-light-current characteristics of the disordered hyperuniform laser compared to a standard MM laser operating in the same operating conditions of Fig. 3 a). The threshold current density J_{th} is reduced from 575 Acm^{-2} for a standard MM laser to 323 Acm^{-2} for this device. The current density of the disordered hyperuniform device has been calculated assuming the nominal area of the active region.

an ultra–low reduction in the current threshold, which results 56% lower than in standard MM device, as low as 323 Acm^{-2}. In our opinion, the former value for the current density threshold reported in the main text represents a lower limit and the value calculated here the upper one.
